# Supplementary material for: Respiratory symptoms and respiratory deaths: A multi-cohort study with 45 years observation time
Source: PLoS One. 2021 Nov 22;16(11):e0260416. doi: 10.1371/journal.pone.0260416 (PMC8608323; doi:10.1371/journal.pone.0260416)
Supplement: S1 Table — (PDF) [file pone.0260416.s002.pdf]

S1 Table. Questions (Q) on respiratory symptoms and scores

**Respiratory symptom group**	**Questions, score**
Cough and phlegm (Bronchitis like symptoms), scores 0-5	Q 8. Do you usually cough and clear your throat in the morning? Yes=1, no=0.
	Q 9. Do you usually cough during the day? Yes=1, no=0.
	Q 10. When you cough or clear your throat do you usually bring up phlegm? Yes=1, no=0.
	Q 11. Do you have cough for 3 months or more altogether during a year? Yes=1, no=0.
	Q 12. During the last 2 years, have had a cough and/or phlegm in connection with a cold for more than 3 weeks? Yes=1, no=0.
Attacks of breathlessness and wheeze (Asthma like symptoms), scores 0-2	Q 17. Do you have attacks of breathlessness? Yes=1, no=0.
	Q 18.Have you ever had wheezing in your chest? Yes=1, no=0.
Breathlessness, scores 0-4	Q 13. Are you more breathless than people of your own age when walking uphill? Yes=1, no=0.
	Q 14. Are you breathless when you climb two flights of stairs at an ordinary pace? Yes=1, no=0.
	Q 15. Are you breathless when you walk on level ground at an ordinary pace? Yes=1, no=0
	Q 16. Are you breathless when at rest? Yes=1, no=0
