# Supplementary material for: Respiratory symptoms and respiratory deaths: A multi-cohort study with 45 years observation time
Source: PLoS One. 2021 Nov 22;16(11):e0260416. doi: 10.1371/journal.pone.0260416 (PMC8608323; doi:10.1371/journal.pone.0260416)
Supplement: S2 Table — (PDF) [file pone.0260416.s003.pdf]

S2 Table. Descriptive statistics for participants at different stages according to response to questionnaire.

	All (response+ nonresponse), with start date*		Response to smoking		Response to education, smoking status		Previous column + response to occupational exposure		Previous column + response to respiratory symptoms	
	No.	%	No.	%	No.	%	No.	%	No.	%
Age, years										
15-29	18212	12	12783	11	12391	11	12083	11	11832	11
30-44	59158	38	40417	35	39511	35	38561	36	37817	36
45-59	42985	27	32617	29	31985	29	30640	29	29613	29
≥60	36541	23	28563	25	28237	25	26191	24	24619	24
Sex										
Male	122734	78	89143	78	87614	78	84361	78	81510	78
Female	34162	22	25237	22	24510	22	23114	22	22371	22
Highest attained education										
Compulsory education (<11 years)	39966	26	29513	26	29513	26	27354	25	25950	25
Medium level (11-13 years)	77920	52	58200	52	58200	52	56140	52	54390	52
University level (>13 years)	33288	22	24411	22	24411	22	23981	22	23541	23
Smoking status										
Never			38699	34	37774	34	36103	34	34916	34
Previous			30676	27	30214	27	28993	27	27986	27
Current			45005	39	44136	39	42379	39	40979	39
No. of cigarettes per day										
0-9			16521	24	16229	24	15502	24	14957	24
10-19			34238	50	33710	50	32510	50	31583	50
≥20			18140	26	17755	26	17230	26	16682	26
Occupational exposure gas/dust										
Yes			48376	44	47509	44	47509	44	45563	44
No			61232	56	59966	56	59966	56	58318	56
Cohort study										
Oslo 72	19892	13	17680	15	17377	15	16445	15	16084	15
Hordaland 85	4982	3	4404	4	4347	4	4307	4	4137	4
Støvlunge 88-90	108812	69	76673	67	75406	67	71958	67	69168	67
Oslo/Hordaland 98-99	23210	15	15623	14	14994	13	14765	14	14492	14
N	156896		114380		112124		107475		103881	

* education was only available for n=151174
