# Supplementary material for: Respiratory symptoms and respiratory deaths: A multi-cohort study with 45 years observation time
Source: PLoS One. 2021 Nov 22;16(11):e0260416. doi: 10.1371/journal.pone.0260416 (PMC8608323; doi:10.1371/journal.pone.0260416)
Supplement: S3 Table — (PDF) [file pone.0260416.s004.pdf]

S3 Table. Prevalence of baseline symptoms and distribution of symptoms according to principal respiratory cause of death.

		Symptoms at baseline		Respiratory disease deaths		Lung cancer deaths		COPD deaths		Pneumonia/ influenza deaths	
		No.	%	No.	%	No.	%	No.	%	No.	%
No. of breathlessness symptoms											
	0	83444	80	3825	64	1672	68	831	48	1039	77
	1	10747	10	859	14	349	14	307	18	142	11
	2	6706	6	736	12	281	12	314	18	101	7
	3	2207	2	362	6	108	4	169	10	47	3
	4	777	1	167	3	32	1	96	6	19	1
No. of cough/phlegm symptoms											
	0	67456	65	2836	48	1140	47	577	34	877	65
	1	18746	18	1147	19	487	20	361	21	223	17
	2	7667	7	705	12	320	13	224	13	118	9
	3	4577	4	470	8	206	8	183	11	60	4
	4	3089	3	422	7	150	6	196	11	39	3
	5	2346	2	369	6	139	6	176	10	31	2
No. of asthma/wheeze symptoms											
	0	78084	75	3605	61	1493	61	776	45	1058	78
	1	18481	18	1509	25	670	27	543	32	215	16
	2	7316	7	835	14	279	11	398	23	75	6
Total		103881	100	5949	100	2442	100	1717	100	1348	100
