# Supplementary material for: Respiratory symptoms and respiratory deaths: A multi-cohort study with 45 years observation time
Source: PLoS One. 2021 Nov 22;16(11):e0260416. doi: 10.1371/journal.pone.0260416 (PMC8608323; doi:10.1371/journal.pone.0260416)
Supplement: S4 Table — (PDF) [file pone.0260416.s005.pdf]

S4 Table. Hazard ratios (HR) with 95% confidence intervals and p-values for lung cancer death according to subgroup, multivariable proportional hazards regression analysis.

		Men		Women		Never smokers		Without cardiopulmonary disease	
		HR	95%CI	HR	95%CI	HR	95%CI	HR	95%CI
Highest attained education									
Medium level (11-13 years) vs. compulsory education (<11 years)		0.56***	[0.51,0.61]	0.47***	[0.37,0.60]	0.46***	[0.30,0.71]	0.66***	[0.57,0.76]
University level (>13 years) vs. compulsory education (<11 years)		0.26***	[0.22,0.31]	0.20***	[0.13,0.31]	0.28***	[0.16,0.49]	0.30***	[0.23,0.40]
Smoking status									
Previous vs. never		4.85***	[3.79,6.21]	1.87*	[1.08,3.26]			4.24***	[2.92,6.16]
Current vs. never		12.05***	[9.53,15.24]	6.91***	[4.79,9.98]			9.37***	[6.57,13.38]
Occupational exposure gas/dust									
No vs. yes		1.19***	[1.09,1.31]	1.35	[0.98,1.87]	1.3	[0.84,2.02]	1.12	[0.96,1.31]
Breathless on effort, score									
	1 vs. 0	1.27***	[1.12,1.44]	0.96	[0.68,1.36]	1.34	[0.64,2.84]	1.23*	[1.01,1.50]
	2 vs. 0	1.93***	[1.67,2.22]	1.35	[0.92,1.98]	2.50*	[1.19,5.22]	1.94***	[1.60,2.36]
	3 vs. 0	2.63***	[2.10,3.29]	2.47***	[1.48,4.13]	3.72*	[1.11,12.51]	2.83***	[2.19,3.65]
	4 vs. 0	1.81**	[1.21,2.70]	1.11	[0.44,2.83]	5.74*	[1.23,26.78]	1.66*	[1.07,2.57]
Cough and phlegm, score									
	1 vs. 0	1.21**	[1.07,1.36]	1.47*	[1.10,1.97]	0.68	[0.37,1.26]	1.18	[0.97,1.43]
	2 vs. 0	1.67***	[1.45,1.92]	1.49*	[1.02,2.18]	1.48	[0.66,3.34]	1.50***	[1.19,1.88]
	3 vs. 0	1.75***	[1.48,2.07]	1.42	[0.89,2.26]	1.05	[0.32,3.46]	1.62***	[1.26,2.08]
	4 vs. 0	1.70***	[1.39,2.07]	1.88*	[1.14,3.12]	0	[0.00,0.00]	1.58***	[1.21,2.08]
	5 vs. 0	2.12***	[1.72,2.62]	1.72	[0.98,3.01]	1.71	[0.38,7.58]	1.75***	[1.33,2.31]
Attacks of breathlessness and wheeze, score									
	1 vs. 0	1.04	[0.94,1.16]	1.16	[0.86,1.55]	1.2	[0.65,2.22]	1.12	[0.95,1.33]
	2 vs. 0	0.94	[0.80,1.10]	1.3	[0.88,1.92]	0.91	[0.33,2.53]	0.96	[0.78,1.18]
Sex									
Female vs. male						1	[0.61,1.64]	0.62***	[0.49,0.77]
N		81510		22371		34916		26723	

* p<0.05, ** p<0.01, *** p<0.001
