# Supplementary material for: Respiratory symptoms and respiratory deaths: A multi-cohort study with 45 years observation time
Source: PLoS One. 2021 Nov 22;16(11):e0260416. doi: 10.1371/journal.pone.0260416 (PMC8608323; doi:10.1371/journal.pone.0260416)
Supplement: S5 Table — (PDF) [file pone.0260416.s006.pdf]

S5 Table. Hazard ratios (HR) with 95% confidence intervals and p-values for COPD death according to subgroup, multivariable proportional hazards regression analysis.

		Men		Women		Never smokers		Without cardiopulmonary disease	
		HR	95%CI	HR	95%CI	HR	95%CI	HR	95%CI
Highest attained education									
Medium level (11-13 years) vs. compulsory education (<11 years)		0.47***	[0.43,0.53]	0.44***	[0.34,0.57]	0.35***	[0.24,0.52]	0.52***	[0.45,0.60]
University level (>13 years) vs. compulsory education (<11 years)		0.20***	[0.16,0.26]	0.11***	[0.06,0.21]	0.10***	[0.05,0.20]	0.19***	[0.14,0.26]
Smoking status									
Previous vs. never		3.65***	[2.82,4.72]	1.70*	[1.05,2.73]			2.37***	[1.82,3.08]
Current vs. never		5.70***	[4.46,7.28]	3.09***	[2.22,4.31]			3.02***	[2.35,3.89]
Occupational exposure gas/dust									
No vs. yes		1.49***	[1.34,1.67]	0.73*	[0.55,0.98]	1.83**	[1.19,2.81]	1.43***	[1.23,1.67]
Breathless on effort, score									
	1 vs. 0	2.05***	[1.77,2.37]	1.43	[0.98,2.08]	3.62***	[2.11,6.24]	2.05***	[1.68,2.51]
	2 vs. 0	3.45***	[2.95,4.03]	3.26***	[2.30,4.61]	6.04***	[3.47,10.50]	3.83***	[3.16,4.63]
	3 vs. 0	6.69***	[5.48,8.16]	4.70***	[2.97,7.43]	7.48***	[3.49,16.05]	6.05***	[4.82,7.60]
	4 vs. 0	7.50***	[5.78,9.73]	3.95***	[2.15,7.24]	9.94***	[4.17,23.67]	6.53***	[4.93,8.65]
Cough and phlegm, score									
	1 vs. 0	1.72***	[1.49,2.00]	1.36	[0.97,1.90]	0.95	[0.57,1.58]	1.51***	[1.23,1.85]
	2 vs. 0	2.16***	[1.81,2.58]	1.09	[0.70,1.71]	0.52	[0.18,1.46]	1.73***	[1.36,2.19]
	3 vs. 0	2.54***	[2.09,3.09]	1.43	[0.89,2.29]	1.15	[0.51,2.63]	1.80***	[1.39,2.34]
	4 vs. 0	3.35***	[2.74,4.08]	1.94**	[1.19,3.16]	1.71	[0.76,3.81]	2.95***	[2.32,3.75]
	5 vs. 0	3.26***	[2.63,4.06]	1.72*	[1.03,2.87]	2.78*	[1.24,6.25]	2.57***	[1.99,3.31]
Attacks of breathlessness and wheeze, score									
	1 vs. 0	1.27***	[1.11,1.45]	1.72***	[1.25,2.37]	2.53***	[1.54,4.15]	1.32**	[1.11,1.58]
	2 vs. 0	1.54***	[1.31,1.81]	2.40***	[1.64,3.52]	4.35***	[2.45,7.72]	1.33**	[1.09,1.62]
Sex									
Female vs. male						0.94	[0.59,1.51]	0.60***	[0.48,0.74]
N		81510		22371		34916		26723	

* p<0.05, ** p<0.01, *** p<0.001
