# Supplementary material for: Respiratory symptoms and respiratory deaths: A multi-cohort study with 45 years observation time
Source: PLoS One. 2021 Nov 22;16(11):e0260416. doi: 10.1371/journal.pone.0260416 (PMC8608323; doi:10.1371/journal.pone.0260416)
Supplement: S6 Table — (PDF) [file pone.0260416.s007.pdf]

S6 Table. Hazard ratios (HR) with 95% confidence intervals and p-values for death from pneumonia according to subgroup, multivariable proportional hazards regression analysis.

	Men		Women		Never smokers		Without cardiopulmonary disease	
	HR	95%CI	HR	95%CI	HR	95%CI	HR	95%CI
Highest attained education								
Medium level (11-13 years) vs. compulsory education (<11 years)	0.38***	[0.34,0.44]	0.36***	[0.28,0.45]	0.30***	[0.24,0.37]	0.43***	[0.35,0.53]
University level (>13 years) vs. compulsory education (<11 years)	0.15***	[0.12,0.19]	0.09***	[0.05,0.14]	0.10***	[0.07,0.14]	0.15***	[0.10,0.23]
Smoking status								
Previous vs. never	1.46***	[1.24,1.72]	0.78	[0.55,1.10]			1.31*	[1.01,1.69]
Current vs. never	0.9	[0.76,1.07]	0.53***	[0.41,0.68]			0.69**	[0.52,0.90]
Occupational exposure gas/dust								
No vs. yes	1.72***	[1.51,1.96]	1.15	[0.81,1.63]	1.71***	[1.34,2.17]	1.46***	[1.16,1.82]
Breathless on effort, score								
1 vs. 0	1.47***	[1.20,1.80]	0.75	[0.49,1.14]	1.34	[0.94,1.91]	1.32	[0.99,1.78]
2 vs. 0	2.03***	[1.60,2.58]	0.88	[0.54,1.42]	1.67*	[1.11,2.50]	1.49*	[1.08,2.07]
3 vs. 0	3.15***	[2.17,4.56]	2.32**	[1.31,4.10]	1.75	[0.81,3.80]	3.61***	[2.50,5.23]
4 vs. 0	2.69**	[1.35,5.35]	2.85**	[1.40,5.82]	3.21**	[1.35,7.62]	2.92***	[1.62,5.25]
Cough and phlegm, score								
1 vs. 0	1.23*	[1.03,1.45]	0.73	[0.53,1.02]	0.91	[0.68,1.21]	0.82	[0.62,1.09]
2 vs. 0	1.63***	[1.29,2.06]	1.16	[0.76,1.78]	1.59*	[1.03,2.47]	1.31	[0.92,1.85]
3 vs. 0	1.68***	[1.24,2.27]	0.65	[0.32,1.30]	1.26	[0.68,2.33]	1.22	[0.79,1.86]
4 vs. 0	1.32	[0.88,1.98]	1.4	[0.74,2.64]	0.84	[0.30,2.29]	1.39	[0.87,2.21]
5 vs. 0	1.46	[0.92,2.32]	1.32	[0.65,2.71]	2.70*	[1.24,5.91]	1.33	[0.80,2.21]
Attacks of breathlessness and wheeze, score								
1 vs. 0	0.73***	[0.61,0.88]	1.1	[0.79,1.53]	0.9	[0.65,1.26]	0.86	[0.67,1.10]
2 vs. 0	0.53***	[0.38,0.72]	1.11	[0.67,1.85]	0.63	[0.35,1.16]	0.56**	[0.39,0.79]
Sex								
Female vs. male					1.35*	[1.05,1.72]	0.85	[0.63,1.14]
N	81510		22371		34916		26723	

* p<0.05, ** p<0.01, *** p<0.001
