# Supplementary material for: Respiratory symptoms and respiratory deaths: A multi-cohort study with 45 years observation time
Source: PLoS One. 2021 Nov 22;16(11):e0260416. doi: 10.1371/journal.pone.0260416 (PMC8608323; doi:10.1371/journal.pone.0260416)
Supplement: Attachment — Submitted filename: Rebuttal_letter__PlosOne27sep2021_ks.docx [file pone.0260416.s008.docx]

Comments to editor and reviewers’ comments

Editor:
1. Adherence with PLOS ONE's style requirements, including those for file naming.

**Checked and should comply.**

The PLOS ONE style templates can be found at

[https://journals.plos.org/plosone/s/file?id=wjVg/PLOSOne_formatting_sample_main_body.pdf](https://mail.uio.no/owa/redir.aspx?REF=4C57mV3zuCq4CcVhNtPFCGQIb6J32ykE4S1v0BfkscR9HenK6m3ZCAFodHRwczovL2pvdXJuYWxzLnBsb3Mub3JnL3Bsb3NvbmUvcy9maWxlP2lkPXdqVmcvUExPU09uZV9mb3JtYXR0aW5nX3NhbXBsZV9tYWluX2JvZHkucGRm) and

[https://journals.plos.org/plosone/s/file?id=ba62/PLOSOne_formatting_sample_title_authors_affiliations.pdf<about:blank](https://mail.uio.no/owa/redir.aspx?REF=0xcb6woveHChY_HLCrXhGx5t25yHPihclq12jRpcTjp9HenK6m3ZCAFodHRwczovL2pvdXJuYWxzLnBsb3Mub3JnL3Bsb3NvbmUvcy9maWxlP2lkPWJhNjIvUExPU09uZV9mb3JtYXR0aW5nX3NhbXBsZV90aXRsZV9hdXRob3JzX2FmZmlsaWF0aW9ucy5wZGY8YWJvdXQ6Ymxhbms.)>


2. Updated Competing Interests section:

***"I have read the journal's policy and the authors of this manuscript have the following competing interests: RN reports grants and personal fees from AstraZeneca, grants from GlaxoSmithKline, grants from Boehringer Ingelheim, outside the submitted work. All other authors have declared that no competing interests exist. This does not alter our adherence to PLOS ONE policies on sharing data and materials***.

If there are restrictions on sharing of data and/or materials, please state these. Please note that we cannot proceed with consideration of your article until this information has been declared.

**See below.**


3. Data availability

**We are in general in favor of sharing data sets. However, the baseline data are old and lack the necessary permissions for sharing the data. There are restrictions on the sharing of dataset from the ethics committee and the registries, and in these historical cohorts, the participants have not given consent to sharing and export of data abroad.**

**Ethical committees in Norway were established in 1985, i.e. after some of the cohorts in this study were established. The current study had approval from the Regional ethics committee (Health Region West) in 2007 (No. 054.07-07/4043) with an update in 2017 (No. 2017/1679/REK vest). In addition, there are approvals from the Norwegian Directorate of Health (No. 07/948), The Data inspectorate (07/00414-4/HPG) and Statistics Norway in 2007 (SSB-ref: 07/1667) with an update in 2009 and 2017 (SSB-ref: 09/1245). Several of the approvals have phrases limiting sharing of the data.**

**In 2017, after linkage with variables from Statistics Norway, we have signed a statement from Statistics Norway, whereby we in this project are obliged to not pass information on to persons within or external to the University of Oslo and University of Bergen.**

**Therefore, we have indicated that data from this study are available upon request. As stated, we can make a de-identified data set with the relevant variables available for validation and replication of all study findings. Data request can be sent to the first author.**

[http://journals.plos.org/plosone/s/data-availability#loc-unacceptable-data-access-restrictions](https://mail.uio.no/owa/redir.aspx?REF=zbzYvHa0A_RuHJcqcm-RCMWD-rTwTslXCS_4n8LoBxp9HenK6m3ZCAFodHRwOi8vam91cm5hbHMucGxvcy5vcmcvcGxvc29uZS9zL2RhdGEtYXZhaWxhYmlsaXR5I2xvYy11bmFjY2VwdGFibGUtZGF0YS1hY2Nlc3MtcmVzdHJpY3Rpb25z).

**Comments to reviewers' comments:**

Reviewer #1:

The study by Stavem et al. attempts to establish an association between respiratory symptoms and death from respiratory causes (including pneumonia, lung cancer, COPD, asthma, and other pulmonary diseases) in subjects over 45 years of age. However, the study has several shortcomings that seriously undermine the overall premises on which the conclusions have been drawn or the title has been framed for the reported study.

**There is clearly a misunderstanding here, the paper has a follow-up period of 45 years. (It does not deal with subjects over 45 years of age). The title of the paper, and the abstract has been revised to clarify this.**

Major concerns:

The overall study design is not conforming to its title or the conclusions drawn. What is of particular concern is that -
(i) presence of possible or associated comorbidities in the subjects have not been considered which makes it questionable on whether the recorded deaths apparently from ‘respiratory causes’ can be attributed exclusively to respiratory symptoms

**Regarding the consideration of comorbidities, see comment to (iii), below. In addition, we would like to specify that in the Mortality statistics, the recorded cause of death is the underlying cause of death. Therefore, the recorded deaths had respiratory causes as underlying cause, although the causes may have played a part or been the immediate cause of death. That the coding concerns the underlying cause of death has been clarified in the methods section of the revised manuscript and an additional reference to the data quality of the Norwegian Cause of Death Register (p.7, para 4) and in the heading of Table 2.**

(ii) The study is significantly gender biased (84% male vs. 16% female) with a mean baseline age of 46.8 years which is extremely skewed for a cohort representing subjects above the age of 45 years. In fact, the basis for inclusion of 12% subjects in the age group of 15-44 years in a study aiming to examine respiratory symptoms related mortality in subjects above the age of 45 years is not only unclear but this contributes to the above aberrant effect on the age statistics as well as unnecessarily introduces complications in the data analysis because the factors governing respiratory diseases among such young group of subjects and the relatively aged group of 45 plus that the study tends to represent are extremely different.

**As stated above, the paper does not examine mortality in subjects above 45 years of age; hence this is clearly a misunderstanding.**

**Added to the discussion (p 22, para 5):**

***The largest sub-cohort included mostly only men, hence the pooled sample had relatively few women and may represent a bias, although we adjusted for sex in the analysis. In a previous report, however, we have shown a similar pattern of association between respiratory symptoms and all-cause mortality for men and women in stratified analyses [6].***

(iii) Moreover, BMI, vitamin D deficiency, genetic factors, history of allergy and infections, etc., can also play a determining role in the development of respiratory symptoms and associated mortality. Unfortunately, there is no consideration or mention of these factors in the study

**Added to the discussion (p. 22, para 3:**

***Some limitations of the study should be noted. In this large, pooled sample, we had access to only a limited number of common/harmonized covariates. It is possible that covariates such as cardiovascular risk factors, body mass index, genetic factors, more detailed history of allergy, infections, comorbidities or other variables may be confounders in the relationship between respiratory symptoms and the specific respiratory deaths. During such a long observation time, covariates may also change over time, however, this study only had available covariates at baseline.***

(iv) The data used in the study have been collected between the year 1972-1999 and all subjects were born between 1902 to1973. There are thus grounds to suppose that the study methodologies and techniques used for assessment of the defined respiratory symptoms have not been consistent over such a long period

**Added to the discussion (p22, para 1):**

***The items on respiratory symptoms were identical in all these cohorts, and they were administered by postal questionnaires. All studies had the same primary investigator.***

(v) The pathophysiological dynamics of various pulmonary diseases vary widely especially when parameters like cough and phlegm are used as ‘respiratory symptoms’. Thus, a more uniform and dependable approach like spirometric assessment of respiratory performance (eg. FEV1 and FVC) would have perhaps been more valuable for a study of this nature.

**Added to the discussion (p 22, para 4):**

***Spirometry would have been a useful supplement to subjective perceptions of respiratory symptoms, but that was only available for a fraction of the participants in the pooled sample. Also, spirometry is more resource-consuming than assessment of simple respiratory symptoms in clinical practice. We therefore believe that more knowledge of the prognostic effect of respiratory symptoms with regard to mortality will be of potentially great value in clinical practice.***

Minor concerns:

1. There have been several studies that have examined the relation between respiratory symptoms and mortality in specific age groups. The manuscript fails to critically portray these studies in sufficient detail and thereby enlighten how and why the present study is different from similar studies of the past (e.g. Busselton Health Study by Knuiman et.al.)

**We are well aware of the Busselton study. The Busselton study investigated the association between respiratory symptoms and respiratory deaths, except lung cancer which was grouped with cancers, with 20-26 years of follow-up. The study dichotomized the respiratory symptoms, whereas the present study had graded responses. The Busselton study included forced expiratory volume in 1 second (FEV_1)_ and several cardiovascular risk factors in the analysis of about 4300 subjects, in contrast to the present study with >100,000 subjects that included occupational exposure and education in the analysis, but no spirometry variable. The present study was far larger and had longer observation time than that study, which enabled the analysis of subgroups of respiratory deaths, such as lung cancer, COPD, pneumonia, and less common respiratory diseases as causes of death.**

**We have added a comment in the discussion with more detailed comparison with the Busselton study (p. 20, para 4).**

2. The authors express restrictions on data availability. The scope of such restrictions should have been defined in more detail as availability of all raw data related to studies of the present nature is somewhat essential.

**See comments to the editor (above).**

In summary, the present study lacks sound design and critical supportive data to conclusively establish a relation between respiratory symptoms and mortality especially in subjects over the age of 45. Overall, the study fails to contribute any substantive knowledge or information that is new or valuable to our present understanding of the factors underlying respiratory diseases and related mortality.

**As mentioned above, this is a misunderstanding, as the study has never been limited to subjects > 45 years of age, but analysed symptoms and mortality with a follow-up period of 45 years. What is new in this study is that this study comprises a very large pooled cohort with a very long follow-up period, enabling us to present new data on cause-specific deaths. This would not be possible or meaningful in smaller cohorts.**

Reviewer #2:

In this cohort study, the authors evaluated the association of respiratory symptoms with mortality due to respiratory causes in Norway by leveraging population based health outcome data (National Cause of Death Registry) and cross sectional symptom surveys in 1972, 1985, 1988, and 1998. Although the authors did not make all data underlying their manuscript fully available, their rationale and a pathway for access to this data to confirm findings is reasonable. I’ve included my comments below

- Infection related pneumonia is not generally considered a chronic respiratory condition as mentioned in the beginning of the introduction. If you are referring to interstitial/histiopathologic pneumonia (such as UIP, EIP, etc)., please clarify this in the introduction.

**Agree completely; this sentence in the introduction has been revised to reflect that pneumonia is not chronic disease (p. 3, para 1).**

- What was the temporal association between symptoms and deaths due to pneumonia? Given the observation of increased breathlessness and death due to pneumonia, and the presumption that all deaths did not occur shortly after the survey, is it possible there was another intermediate factor that increased the risk of death due to pneumonia (such as undiagnosed chronic lung disease, bronchiectasis etc)?

**In this study, we used age and not time in study as the time scale in our Cox regression, as recommended by several authors. We assessed the proportional hazard assumption, which was found to be acceptable.**

**However, the reviewer here asks about the association with time in the study. To shed more light on the temporal association with time in study, we have enclosed a Kaplan Meier failure plot for information, but have not included this in the manuscript.**

**In addition, we have added a short comment on pneumonia to the discussion (p. 23, para 3):**

***Pneumonia is a difficult outcome based on cause of death coding, and there might be different predisposing conditions. It is possible that deaths coded as pneumonia as underlying cause of death could reflect unrevealed chronic lung diseases, or be related to other covariates than those that were available in the pooled sample in these cohorts.***

***.***

- Please address the following limitations: Male biased cohort, limitations associated with survey data regarding selection bias.

**This has been added to the discussion:**

- **Male bias: see comment to Reviewer #1 above (p 22, para 3)**
- **As in any survey, there is a risk of non-participation bias; however, it is hard to delineate how this would impact the association between respiratory symptoms and the studied outcome. The response rate in the surveys in this study ranged 67–89%, which is higher than what would probably be achievable today, as survey response rate to epidemiological studies have decreased over the past decades [25]. However, response rates depend on the topic and length of the questionnaire, target population, and whether incentives are used [25-27]. (p. 22, last paragraph)**

Reviewer #3: First, a caveat. On the surface, the methods seem OK, but my expertise is dyspnea mechanisms and measurement, not epidemiologic stats. I am not equipped to make a serious critique of epidemiologic statistical methods in this study.

This analysis utilizes data collected in 4 previous cross sectional studies conducted between 1972 and 1990 in Norway utilizing a Norwegian adaptation of the MRC respiratory questionnaire in 100,000 people. The authors examine the relationship between respiratory symptoms and cause of death. Of the eligible subjects (born between 1902 and 1973), 44,000 had died by the end of 2016 when data collection was terminated. This is a pretty heterogeneous group – subjects would have been 43 to 114 years of age at the study termination, and there would have been a wide variation in the time between survey and death. The main finding of the paper is that subjects who reported respiratory symptoms during a population survey are more likely to die from respiratory disease than subjects who do not report respiratory symptoms. The sample was then subcategorized by respiratory disease (COPD, lung cancer, pneumonia …). The strongest association was between COPD as cause of death and reported breathlessness (increasing breathlessness score associated with increasing hazard ratio - a breathlessness score of 4 was associated with a 7-fold risk of COPD death). These findings are unsurprising to those familiar with the literature.

The information in this paper may be useful to some, but it would benefit from a less narrow view. I am in agreement with the authors’ conclusion that “Health personnel should be encouraged to ask patients about their symptom burden, as this may be a simple and cost-effective way to map increased mortality risk also among those without known illness. Awareness of even common respiratory symptoms is important in detecting deadly underlying diseases.” In fact, I have been supporting this idea for years. There is, however, substantial published information supporting this idea (much of which has not been cited by the authors). I’m not sure the present work, in its current form, adds much. Perhaps the authors could consider enhancing its usefulness.

**We have added a paragraph in the discussion to address this (p. 20, para 3):**

***This study comprises a very large pooled cohort with a very long follow-up period, enabling us to present new data on cause-specific deaths. This is a strength of the present study and would not be possible or meaningful in smaller cohorts and explains why there is little previous information on the association between respiratory symptoms and the less common causes of respiratory deaths.***

1) The paper has a narrow focus on a particular epidemiologic question, and lacks broader context. There are several sources for broader information on dyspnea and cough. You could fit this together with your observations – why for instance, would dyspnea be such a powerful predictor of COPD death, but a much weaker predictor of lung cancer and pneumonia death? Why does it predict pneumonia death at all? Pneumonia is an acute disease. These people were not surveyed as part of a medical encounter, so they would be unlikely to have pneumonia symptoms at the time of survey. Is the connection indirect, through COPD? One might also consider the measurement circumstances and timing – what was the gap between survey and death? What was the age at death, etc. Also one might consider the physiologic mechanisms causing cough and breathlessness – how are these related to disease process, and does this inform the interpretation of the link between symptom and cause of death? The questionnaire used (a revision of the MRC) is quite old (consistent with the date of the data collection) – how does that limit the study? Do you think more modern instruments would improve sensitivity or specificity? Why? Or why not?

**This was an epidemiological study designed to investigate potential associations. We do not have data to highlight physiological mechanisms, therefore this would be hypothetical or speculative.**

**Yes, the MRC questionnaire is more than 60 years old; however, a modified version of the dyspnea questionnaire (mMRC dyspnea questionnaire) is still in common use, e.g. is listed in the most recent GOLD guideline for COPD** [**https://goldcopd.org/wp-content/uploads/2020/11/GOLD-REPORT-2021-v1.1-25Nov20_WMV.pdf**](https://goldcopd.org/wp-content/uploads/2020/11/GOLD-REPORT-2021-v1.1-25Nov20_WMV.pdf)

**and is well documented. There is little documentation on the association of other more recent respiratory questionnaires (e.g. the Baseline dyspnea index (BDI), Dyspnea-12, or PROMIS dyspnea module: The PROMIS SF v1.0 – Dyspnea-Severity 10a, or FACIT-Dyspnea) with mortality in general populations, although some of these might have better psychometric properties than the mMRC, or the version used in the present study. In contrast, there is some documentation on an association with mortality for broader heath status instruments in selected populations with chronic disease, e.g. in COPD (Chronic Respiratory Questionnaire, St. George’s Respiratory Questionnaire, COPD assessment test; CAT), as well as composite scores, such as the BODE index (*Celli BR, et al. N Engl J Med2004;350:1005–12)* for prognostication in COPD, but these are very different cohorts and beyond the scope of the present study.**

2) Can you argue the advantages to the clinician or to public health agencies of routinely assessing and documenting respiratory symptoms? It is not at all surprising or new that respiratory symptoms are associated with respiratory deaths. But are there advantages not elucidated in the paper? Do symptoms predict mortality better than other common indicators in your database? For example look at the key paper by Nishimura (Chest 2002) showing that dyspnea is a better predictor of COPD mortality than FEV1. Do symptoms predict mortality before other common indicators? (time from first report of symptoms to death may be within your database, but is not reported). Other important papers relating respiratory symptoms to mortality are missing from your references and discussion (eg, Abidov NEJM 2005, Santos PLOS One 2016).

**The sample in this study was a large general population and enabled subcategorization of respiratory deaths, in contrast to most previous studies. Smaller studies will have more independent variables but more crude outcomes.**

**This paper is about the association between respiratory symptoms in the general population and respiratory deaths. The mentioned papers deal with other types of cohorts or do not have respiratory outcomes, and have therefore not been considered relevant in this context:**

**Nishimura, Chest 2002: Not general population, but in cohort of patients with COPD. All-cause mortality over 5 years. N=227.**

**Abidov et al. NEJM 2005. Heart stress testing using myocardial-perfusion single-photon-emission computed tomography during stress and at rest. This, again is no general population, but a selected population with known or suspected cardiac disease. Moreover, the outcomes were all-cause mortality and cardiovascular mortality (and not mortality due to respiratory diseases).**

**Santos, PLOS One 2016: No respiratory outcomes, but only cardiovascular outcomes such as incident heart failure, incident coronary heart disease, or all-cause mortality (which are different from the outcomes in the present study.**

3) Dyspnea usually indicates some problem in oxygen delivery and utilization, or problems with CO2 elimination – thus it is associated with many cardiovascular and metabolic diseases in addition to those classified as respiratory disease. Any consideration of dyspnea as a predictor of these other diseases is absent from your work, although these causes of deaths are probably in your database.

**We agree that this would be interesting. The present study focused on respiratory death. In a future paper, we shall investigate the association between respiratory symptoms and sub-categories of cardiovascular deaths.**

4) Information on prevalence of the various scores of the symptoms would be useful – both overall in the sample, and subdivided as in tables S3, S4, S5. By this I mean, for instance, how many people reported a score of 3 on dyspnea, etc.

**We have added a table with the requested information as a supplementary table (new S3 Table).**

5) A curious detail: Why do you suppose that a dyspnea score of 4 was associated with fewer lung cancer deaths than a score of 3?

**A good point, to which we have no clear answer. Dyspnea is subjective, as perceived by the patient. It is possible that 4 (“Are you breathless when at rest?”) also captures e.g. anxiety or hyperventilation as well as respiratory or cardiovascular limitations, whereas 3 (“ Are you breathless when you walk on level ground at an ordinary pace?”) to a larger extent captures respiratory or cardiovascular limitation or deconditioning, which may have a stronger association with respiratory deaths. This is a challenge with the mMRC questionnaire, as well, and possibly the levels 3 and 4 should be combined.**

Reviewer #4:

The research study reported respiratory symptoms and death from respiratory causes. In summary, 14% of the examined population died from respiratory causes. Of these, 41% died from lung cancer, 29% from COPD, 23% from pneumonia, 2% from asthma, 2% from interstitial lung disease and 3% from other pulmonary diseases.

Minor revisions:
1- Line 191: State the proportion of men who died.

**Added the requested information to the text (p. 10, para 5)**

2- Title and Abstract: Clarify that 45 years refers to a span of time, not the participant's ages.

**Thanks, this has been clarified in the revised manuscript. See comment to reviewer #1.**


[NOTE: If reviewer comments were submitted as an attachment file, they will be attached to this email and accessible via the submission site. Please log into your account, locate the manuscript record, and check for the action link "View Attachments". If this link does not appear, there are no attachment files.]

While revising your submission, please upload your figure files to the Preflight Analysis and Conversion Engine (PACE) digital diagnostic tool, [https://pacev2.apexcovantage.com/](https://mail.uio.no/owa/redir.aspx?REF=U5zhPC29dg_6HUlYcLLzTLOYo5segKslJrdG4LIY6L-QROnK6m3ZCAFodHRwczovL3BhY2V2Mi5hcGV4Y292YW50YWdlLmNvbS8.). PACE helps ensure that figures meet PLOS requirements. To use PACE, you must first register as a user. Registration is free. Then, login and navigate to the UPLOAD tab, where you will find detailed instructions on how to use the tool. If you encounter any issues or have any questions when using PACE, please email PLOS at figures@plos.org<[mailto:figures@plos.org](https://mail.uio.no/owa/redir.aspx?REF=y10_rHl-gHot9pN_8ftK98qJu5tvknqzosx3h23AwdyQROnK6m3ZCAFtYWlsdG86ZmlndXJlc0BwbG9zLm9yZw..)>. Please note that Supporting Information files do not need this step.
